# Combined Method to Remove Endotoxins from Protein Nanocages for Drug Delivery Applications: The Case of Human Ferritin

**DOI:** 10.3390/pharmaceutics13020229

**Published:** 2021-02-06

**Authors:** Filippo Silva, Leopoldo Sitia, Raffaele Allevi, Arianna Bonizzi, Marta Sevieri, Carlo Morasso, Marta Truffi, Fabio Corsi, Serena Mazzucchelli

**Affiliations:** 1 Dipartimento di Scienze Biomediche e Cliniche “L. Sacco”, Università di Milano, 20157 Milano, Italy; filippo.silva@unimi.it (F.S.); leopoldo.sitia@unimi.it (L.S.); raffaele.allevi@unimi.it (R.A.); arianna.bonizzi@unimi.it (A.B.); marta.sevieri@unimi.it (M.S.); 2 Istituti Clinici Scientifici Maugeri IRCCS, 27100 Pavia, Italy; carlo.morasso@icsmaugeri.it (C.M.); marta.truffi@icsmaugeri.it (M.T.)

**Keywords:** drug delivery, protein nanocages, endotoxin purification, ferritin

## Abstract

Protein nanocages represent an emerging candidate among nanoscaled delivery systems. Indeed, they display unique features that proved to be very interesting from the nanotechnological point of view such as uniform structure, stability in biological fluids, suitability for surface modification to insert targeting moieties and loading with different drugs and dyes. However, one of the main concerns regards the production as recombinant proteins in *E. coli*, which leads to a product with high endotoxin contamination, resulting in nanocage immunogenicity and pyrogenicity. Indeed, a main challenge in the development of protein-based nanoparticles is finding effective procedures to remove endotoxins without affecting protein stability, since every intravenous injectable formulation that should be assessed in preclinical and clinical phase studies should display endotoxins concentration below the admitted limit of 5 EU/kg. Different strategies could be employed to achieve such a result, either by using affinity chromatography or detergents. However, these strategies are not applicable to protein nanocages as such and require implementations. Here we propose a combined protocol to remove bacterial endotoxins from nanocages of human H-ferritin, which is one of the most studied and most promising protein-based drug delivery systems. This protocol couples the affinity purification with the Endotrap HD resin to a treatment with Triton X-114. Exploiting this protocol, we were able to obtain excellent levels of purity maintaining good protein recovery rates, without affecting nanocage interactions with target cells. Indeed, binding assay and confocal microscopy experiments confirm that purified H-ferritin retains its capability to specifically recognize cancer cells. This procedure allowed to obtain injectable formulations, which is preliminary to move to a clinical trial.

## 1. Introduction

Interest in protein nanocages (P-NCs) has been growing over the past years since they turn out to be fascinating drug delivery systems. Their appeal relies mainly on their minimal toxicity, good biocompatibility and easy metabolism, since they can be degraded following physiological protein degradation routes [1]. Indeed, P-NCs are constituted by protein monomers that self-assemble in hollow structures [1]. P-NCs constitutive monomers can be chemically or genetically modified by inserting surface functionalities, in order to tune surface charge, ligand display, stability and drug loading [2]. Different P-NCs have been developed in the last twenty years such as Vault, viral capsids and Heat-shock proteins, but the H-ferritin nanocage (HFn) represents “The Golden boy” of P-NCs developed for drug delivery [3,4].

HFn is a homopolymer of 24 H-ferritin subunits arranged in a sphere structure of 12 nm in diameter. Its protein shell encloses a uniform-size cavity of 8 nm in diameter, which can accommodate different kinds of drugs but also allows to control the amount of encapsulated molecules, which is a crucial parameter to check in nanoformulation [5,6,7,8,9]. Moreover, HFn folding into its quaternary structure is a pH-dependent reversible process, which allows both to easily perform the loading and to control drug release [3,10]. Furthermore, HFn internal and external surfaces could be chemically or genetically modified by inserting targeting functionalities to improve P-NC biodistribution and/or circulation time [11,12].

In the last twenty years, HFn has found application mainly in the oncological field, helping to improve tumor-targeted drug delivery thanks to its natural tumor homing. Indeed, HFn is internalized in cancer cells upon its specific binding with the Transferrin receptor 1 (TfR1) [13], which is overexpressed in almost all kinds of cancer [14]. As a result of that, HFn has been exploited to develop several HFn-based nanodrugs displaying improved activity due to specific tumor recognition, increased drug penetration and chemosensitivity, and reduced side-effects [15,16,17]. 

Despite the alleged effectiveness of HFn-based nanodrug in oncology, the way for a clinical application is still long and curvy. One of the main issues is represented by the possible presence of endotoxin (ETX) contaminants in the product. Indeed, HFn is produced as a recombinant protein in the Gram-negative bacterium *E. coli* with good purification yields [18]. However, when we move from small to large scale production, there is a great chance that, even after purification, HFn is still contaminated with ETXs, which are known to be immunogenic and pyrogenic [19,20]. Since ETXs (i.e., lipopolysaccharides (LPS)—some of the main components of the bacterial wall) can exert an immune reaction when injected into a living organism, it is mandatory to limit the maximum amount of them that still remain in such HFn after purification [21]. In order to understand how this can be achieved, it is necessary to mention that ETXs concentration is generally extremely low if compared to the concentration of the target protein [22]. Thus, the overall amount of ETX is normally expressed in EU (endotoxin units), where 1 EU approximately corresponds to 0.1/0.2 ng.

As a side note, it is important to mention that ETX contamination does not involve HFn or P-NCs only, since most recombinant proteins are produced in *E. coli* (a gram negative bacterium containing lipopolysaccharides in its external wall), thus requiring a certain level of further purification in order to remove ETX residues. However, getting rid of ETX in small, globular proteins is quite simpler and it was extensively studied with several different methods, which seem not to be so effective on more complex proteins with high molecular weights. HFn, in particular, poses a double challenge as it is a large, multimeric protein whose monomers actually form a nanocage. Removing ETX from HFn is then difficult due to its big dimensions (which result in a higher ETX content) combined with the need to preserve its quaternary structure (that indeed qualifies it as a P-NC).

Since the issue of pyrogenicity should be considered when using P-NCs for in vivo experiments that could eventually lead to clinical trials on humans and patented drugs, several institutions, such as the American Food and Drug Agency (FDA) and the European Medicines Agency (EMA), developed a system to calculate the maximum amount of ETX that can be contained in a formulation in order for it to be approved. Concerning the generally accepted limit for commercial drugs, the value differs according to the route of administration: intravenous route has a limit of 5 EU/kg, whereas for intrathecal administration it is only allowed an ETX value of 0.2 EU/kg. For instance, when performing in vivo experiments in mice via intravenous administration, the formula would require to necessarily respect the limit of 1 EU/mL; therefore, the ETX concentration in the nanoformulation will need to be lower than 1 EU/mL [23,24,25,26].

Some guidelines were defined also regarding the allowed techniques to be used to quantify the amount of ETXs. So far, the best known certified test is the *Limulus Amebocyte* Lysate (LAL) assay, which over the years has been declined in several variations, all based on the same principle: the ability of a particular molecule found in the amebocytes of the Limulus species to react with ETX [27,28]. LAL assay turned out to be effective and offers adequate reproducibility, especially when performing a kinetic turbidimetric assay to determine the sample absorbance at 405 nm over a period of 60 min with repeated readings. LAL test has two main advantages: it is certified as a valid method to assess the presence of ETXs by FDA and, more importantly, it is extremely sensitive. Its disadvantages should also be considered though: readings can be easily compromised by traces of other molecules (e.g., residual Triton) and the whole procedure may become quite expensive, due to the high costs of LAL disposables and reagents [29]. In addition, the use of Limulus-derived lysate poses some ethical issues, as it involves the employment of enzymes that are directly collected from living beings. Several solutions are currently under development to solve the problem: Limulus harvesting is under strict control and every animal, after blood donation, is released at a distance from where it was captured, in order to avoid the same animals to be rebleeded. Moreover, synthetic alternatives to LAL are being studied and tested and might eventually replace LAL, thus stopping the hunt for horseshoe crabs [30,31,32]. 

As a natural consequence of what mentioned so far, there is rising attention to all the procedures able to remove ETXs from P-NCs in order to use them as nanocarriers for drug delivery. However, depyrogenation is not an easy process to adopt on P-NCs, mostly because ETX is not affected by pH or temperature variations and tends to stay attached to the protein without forming aggregates [33].

Conventional methods of purification, such as size-exchange chromatography, appear not to be effective in removing ETXs as they bind proteins too strongly [34]. Several methods are currently available or still being developed to achieve the removal process whilst maintaining a good protein recovery, since protein loss is another main issue of any purification process [35]. 

These purification methods are based on affinity chromatography approaches or the employment of detergents [35]. Available resins used for affinity purification exploit different adsorbents immobilized on a substrate. The most used removal agent is Polymixin B, a cationic surfactant that binds the phosphatidil-etanolammine on bacterial lipopolysaccharide. The real efficacy of these methods is controversial, mostly because it strongly varies depending on the nature of the protein to purify and the overall amount of ETX in the sample [33,36]. 

ETX purification procedures based on the use of detergents employ non-ionic detergents, such as Triton X-100 or Triton X-114. This class of molecules turned out to be effective because they can form a two-phase solution [37]. In the case of Triton X-114 (the detergent we successfully used to develop our protocol), one phase contains most of the ETX-intercalating micelles, since the portion of ETX known as lipid A is bound by the surfactant tail group of Triton and it is separated from the aqueous phase, where the ETX-free proteins remain and can be collected from. Another key feature of Triton X-114 is represented by its cloud point, found to be at 22 °C: above that temperature, micelles tend to aggregate and can be separated by centrifugation, thus allowing the removal of ETX as well [38,39].

Beyond the previously described procedures, ultrafiltration was also proposed as a method to remove ETXs, but it proved to be quite ineffective and dangerous for protein stability [40].

Here, we developed a protocol for the purification of P-NCs and in particular of HFn, whose purification from ETX was not previously studied. We have exploited a two-step procedure involving both resins and detergents, followed by gel filtration to remove residual reagents, in order to make HFn eligible for injection, according to the rules of FDA, both in mice (for in vivo studies) and in human.

## 2. Materials and Methods

### 2.1. Materials

Pyrogen-free tested water (cat #ECM0970L), PBS (cat #ECB4053L), pyrogen-free certified sterile tubes (cat #ET5050B, #ET5015B), cell culture medium Roswell Park Memorial Institute 1640 (RPMI 1640) (cat #ECB9006L), goat serum (cat #ECS0200L), fetal bovine serum (FBS, cat #ECS0170LI), 1.5 mM l-glutamine, 100 U/mL penicillin and 0.1 mg/mL streptomycin, were purchased from Euroclone, Pero, Italy. Triton™ X-114 (cat #X114), fluoresceine isothiocyanate (FITC, cat #F7250), Bovine Serum Albumin (BSA, cat #A7906), paraformaldehyde (cat #158127), and MidiTrap G25 columns (cat # GE28-9180-08) were purchased from Sigma-Aldrich, Merck Life Science, Milano, Italy. Zeba™ Spin Desalting Column 7K MWCO, 2 mL (cat #89890) primary anti-TfR1 antibody (cat #MA1-7657, clone ICO-92), goat anti-mouse Alexa Fluor 488 secondary (cat# A-11001), DAPI (4′,6-diamino-2-phenylindole, cat #D1306), Prolong Gold Antifade Reagent (cat #P10144), Coomassie Protein Assay Reagent (cat #23200), were purchased from Thermo Fisher Scientific Inc., Monza, Italy. 

### 2.2. HFn Nanocages

Recombinant Human apoferritin H-homopolymer (HFn), produced as a recombinant protein in *E. coli,* was purchased from Molirom s.r.l. (Rome, Italy) and stored at 4 °C for the whole duration of experiments. Before using HFn for experiments, protein solution was centrifuged (10′ 10,000× *g*, 4 °C) to remove protein aggregates. Protein concentration in the supernatant was measured by A280 analysis and by Bradford assay (Nanodrop 2000c, Thermo Fisher Scientific Inc., Monza, Italy). 

### 2.3. HFn Endotoxin Removal with EndotrapHD Resins

#### 2.3.1. Column Mode

Endotrap^®^ HD prepacked columns containing 1 mL of resin (LET0010, Lionex GmbH, Braunschweig Germany) were regenerated and equilibrated according to the manufacturer protocol, through three cycles of centrifugation (3000× *g*, 2′) in 1× regeneration and equilibration buffers, using 15 mL pyrogen free certified sterile tubes. HFn was suspended in equilibration buffer at 1 mg/mL, and 3 mL of protein suspension were loaded into the column. To elute HFn and maximize ETX-resin contact time, up to 6 centrifugation steps were included to the manufacturer protocol, testing different centrifugation speed (3000× *g* for 2′ or 100× *g* for 10′), to facilitate HFn passage and increase protein recovery, as reported in Table 1. Centrifuge was initiated as soon as the resin volume was filled with the protein. The centrifugation time of 2′ was chosen according to the instructions by the manufacturer, while 10′ were selected as it was the shortest time that allowed us to recover all the protein volume loaded in the column at that given speed (100 g).

At the end of the protocols, protein recovery has been evaluated by A280, and endotoxin levels have been measured by the kinetic turbidimetric LAL test (for details see below).

#### 2.3.2. Batch Mode

Endotrap^®^ HD resin (Lionex GmbH, Braunschweig Germany) was incubated with HFn in batch mode. New resin was regenerated and equilibrated in 15 mL pyrogen-free certified sterile tubes, as previously described for the column mode. HFn was resuspended in equilibration buffer at 1 mg/mL and the amount of ETX was determined by LAL test, as reported above. Since the resin is able to bind 5 × 10^6^ EU/ mL, HFn solution (1 mg/mL) was incubated with an excess of resin one hundred-fold more than ETX contamination. For instance, one milliliter of HFn solution (1 mg/mL) containing 5 × 10^4^ EU has been incubated with 1 mL of Endotrap HD resin. Indeed, an excess of resin was considered necessary in order to be sure that all the ETX content was bound and removed from the sample. The protein-resin mix was incubated on an orbital shaker at 180 rpm at room temperature (RT) for 2 h. At the end of the incubation the whole protein-resin mix was transferred to an empty column to separate the resin (trapped in the column) from the protein, that was collected into pyrogen-free certified sterile tubes. The resins were washed twice with an amount of equilibration buffer equal to 50% of the recovered protein volume. Protein concentration and endotoxin content of all the collected fractions were measured by A280 and LAL test, respectively. 

### 2.4. HFn Endotoxin Removal with Triton X-114

HFn purification with Triton X-114 was performed under different conditions and the protocol was adjusted to achieve the best results in terms of protein recovery and ETX removal.

Firstly, HFn—at different concentrations: 1 mg/mL, 2 mg/mL, 5 mg/mL, 10 mg/mL in PB —was mixed with Triton X-114 1% (*v/v*) in small aliquots, each with a final volume of 1 mL. The resulting solutions were incubated at 4 °C on a microtube rotator (20 rpm) for 30 min, then moved to 37 °C (without stirring/shaking) for 10 more minutes. At the end of the latter incubation, tubes were centrifuged at 35 °C for 15 min at the maximum available speed (17,000× *g*). After centrifugation, two phases were clearly visible inside the tubes: a thick phase at the bottom, mainly composed by hydrated Triton X-114 and ETX, and an upper phase containing the purified protein in PBS. The upper phase, whose total volume varied from 850 µL to 900 µL, was carefully collected and put in a new tube, then the final volume was reset to 1 mL by adding PBS and fresh Triton X-114, in order to start a new cycle. After completing four cycles, the supernatant was carefully collected (without perturbing the Triton-containing bottom phase) and put into a clean tube for further processing.

Purified HFn underwent a gel filtration procedure to remove residual Triton X-114; the process was performed with MidiTrap G25 columns according to the manufacturer instructions. The resulting HFn was quantified by measuring the absorbance at 280 nm and stored at 4 °C in non-pyrogenic glass tubes.

In order to enhance the purification process, it was also tested an increase in the number of cycles with Triton X-114 (up to six). In the attempt to make the process quicker and more efficient, HFn purification was also performed in a single-step procedure involving a higher amount of Triton X-114 (2%, *v/v*).

### 2.5. Combination of Endotrap HD Resin and Triton X-114

To obtain injectable HFn solutions, a double-step procedure was developed. The first step involved the use of Endotrap^®^ HD resin in batch to roughly lower the amount of ETX contained in the sample. New resin was regenerated and equilibrated according to the manufacturer instructions and incubated with 10 mg/mL concentrated HFn (1:1 *v/v*) in a 15 mL centrifuge tube. Incubation was performed at RT for 2 h on a shaker (150–180 rpm) to maximize the interaction between the resin and ETXs. After completing the incubation phase, the protein-resin solution was transferred to an empty column in order to elute the purified protein. The column was washed with equilibration buffer (1 mL, 3×) and the washing solution was collected in a new tube. The amount of HFn contained in all samples was determined by measuring the absorbance at 280 nm. Due to the results of the quantification, purified HFn was mixed with samples derived from the first and second wash, as their protein concentrations were alike.

An aliquot of the final sample was collected to be analyzed via LAL assay, while the rest of it was divided into smaller samples (1 mL each) that were further purified with the Triton X-114 protocol previously described (four cycles with Triton X-114 1% (*v/v*)). Each fraction was quantified (A280 nm) and its ETX content was evaluated with a LAL assay. After assessing the purity of the protein, all HFn fractions were mixed and stored at 4 °C in non-pyrogenic glass tubes. 

### 2.6. Transmission Electron Microscopy

The morphologies of native and purified HFn were evaluated by transmission electron microscopy (TEM). HFn was diluted to a final protein concentration of 250 µg/mL in mQ H_2_O. A 20 µL drop of suspension was spotted on a Formvar grid and let drying at RT. Then, the grid was stained with the contrast agent uranil-acetate 1% for 30 s at RT and dried O/N at RT. At least 10 images per sample were acquired by TEM (Tecnai Spirit, FEI, Hillsboro, OR, USA) at 80 k–135 k × and 220 k–300 k × magnification. The size profiles of at least 100 HFn molecules were elaborated by ImageJ Profile Plot tool to evaluate whether the ETX purification altered the size distribution of the nanocages.

### 2.7. LAL Test

To test the ETX content in the different protein preparations, it was used the *Limulus Amebocyte* Lysate (LAL) kinetic turbidimetric assay according to the manufacturer instructions (Charles River Microbial Solutions Ltd., Dublin, Ireland). A standard curve was prepared fresh before every analysis by using standard ETX (E120, Charles River Microbial Solutions Ltd., Dublin, Ireland) in the range between 50 and 0.005 EU/mL. Different sample dilutions were prepared in pyrogen-free tested water according to the initial ETX content: the purpose was to obtain samples with a final ETX content falling within this range. Results were considered reliable only when spike recoveries were between 50% and 200% (spike value = 5 EU/mL). The same pyrogen-free tested water used for dilutions served also as blank, while equilibration buffer, PBS, and PBS-Triton X-114 1% (*v/v*) were tested to exclude any interference with the LAL readings.

### 2.8. HFn FITC Preparation

Both native and ETX-free HFn were conjugated with fluorescein isothiocyanate (FITC) according to the manufacturer protocol. Briefly, an excess of FITC dissolved in ethanol (2 mg/mL) was mixed with 0.15 M sodium bicarbonate, before adding HFn at a final concentration of 1 mg/mL (1 mL final volume). The suspension was incubated at 100 rpm on an orbital shaker at RT for 1h. Unconjugated FITC was removed with Zeba™ Spin Desalting Columns. Protein and FITC concentrations were measured by Nanodrop, using the Proteins and Labels protocol (Nanodrop 2000c, Thermo Fisher Scientific Inc., Monza, Italy).

### 2.9. Cell Culture

Immortalized breast cancer murine 4T1-Luc2 (Bioware Ultra, Perkin Elmer, Milan, Italy) and human breast carcinoma HCC1937 (ATCC-LGC Standards, Sesto San Giovanni, Italy) were cultured in RPMI 1640 supplemented with 10% heat inactivated fetal bovine serum (FBS), 2 mM l-glutamine, 100 U/mL penicillin and 0.1 mg/mL streptomycin. Human colon adenocarcinoma HT29 cells (ATCC-LGC Standards, Sesto San Giovanni, Italy) were cultured in RPMI 1640 medium supplemented with 10% heat inactivated FBS, 1.5 mM l-glutamine, 100 U/mL penicillin and 0.1 mg/mL streptomycin. All cell lines were grown at 37 °C in a humidified atmosphere containing 5% CO_2_ and were subcultured prior to confluence using trypsin/EDTA.

### 2.10. Biological Interaction of Purified HFn with Cells

#### 2.10.1. TfR1 Expression on Different Cell Lines

TfR1 expression has been evaluated by flow cytometry, using the CytoFLEX flow cytometer (Beckman Coulter, Cassina De’ Pecchi, Italy). Cells were resuspended at a concentration of 5 × 10^5^ cells/tube in blocking buffer (PBS, 2% Bovine Serum Albumin (BSA) and 2% Goat serum) and pelleted by centrifugation (5′, 300× *g*, RT).

HT29 and HCC1937 were incubated with the murine primary antibody that recognizes human TfR1 (1 µg/tube, 15′ at RT) in blocking buffer. Cells were washed three times with PBS and labelled with goat anti-mouse Alexa Fluor 488 secondary antibody (1:200, 1 µL/tube ; 15′ at RT) in blocking buffer. 4T1-luc cells were incubated with the APC labelled antibody that recognizes murine CD71 (Clone REA627, Miltenyi Biotec S.r.l. Bologna, Italy; cat#:130-119-133), according to the manufacturer protocol. Immunodecorated cells were washed thrice with PBS before analysis. After gating on viable singlet cells, 20,000 events per sample were acquired. Unlabeled cells or immunodecorated with the secondary antibody only were used to set the region of positivity.

#### 2.10.2. Protein Binding and Interactions with Cells

Native and ETX-free HFn interaction with cells was evaluated by flow cytometry. FITC labeled HFn (F-HFn) were incubated with cells (5 × 10^5^ cells/tube) at different concentrations of HFn (10, 20 and 100 µg/mL respectively) in PBS-BSA 0.3%, 2 h at 4 °C in the dark. At the end of incubation cells were washed three times in PBS and analyzed by cytofluorimetry, as previously described.

To further characterize the specificity of the binding, cells were pre-incubated with unlabeled HFn as competitor (HFn 1 mg/mL in 0.3% BSA-PBS, 4°C in the dark). After 1 h of incubation, 20 µg/mL of F-HFn was added to the suspension and incubated for 2 h at 4 °C in the dark. Cells were washed three times with PBS and analyzed by cytofluorimetry, as previously described.

#### 2.10.3. Confocal Microscopy

Cells (2 × 10^5^) were cultured on collagen-coated cover glass slides for 24 h and incubated with F-HFn at a concentration of 100 µg/mL for 6 h at 37 °C. After incubation, cells were washed three times with PBS, fixed for 5 min with 4% paraformaldehyde and washed thrice with PBS. Nuclei were labelled with 0.2 µg/mL DAPI (10′, RT) and cells were washed three more times in PBS. Coverslips were mounted onto Superfrost microscopy glass slides in Prolong Gold antifade reagent. Microscopy analysis was performed with the Leica SP8 confocal microscopy system (Leica Microsystems, Wetzlar, Germany) equipped with laser excitation lines at 405 and 488 nm, using 63× magnification oil immersion lens.

### 2.11. Statistical Analysis

Statistical analyses were conducted using two-tailed Student’s t-test in case of data that passed the Shapiro-Wilk normality test, or with the non-parametric Wilcoxon-Mann-Whitney test in case of non-normal distribution of the data. Results are expressed as means ± standard deviation (S.D.). The statistical significance threshold was set at *p* < 0.05.

## 3. Results

### 3.1. Endotoxin Removal with Endotrap^®^ HD Resins

#### 3.1.1. Column Mode

**Figure 1:**
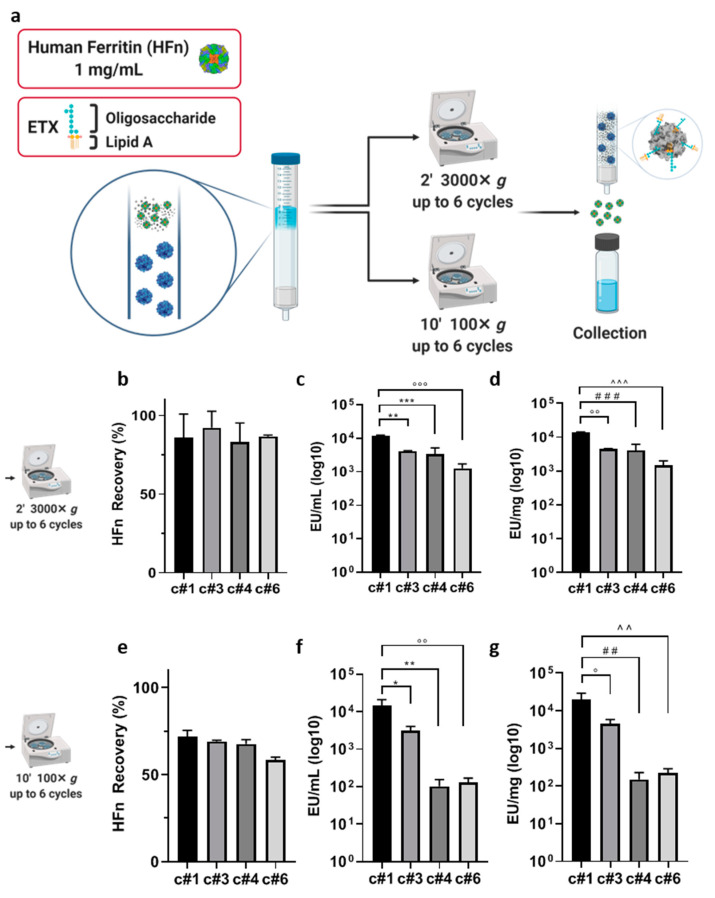
Purification strategy using Endotrap HD columns. The column was loaded with HFn (1 mg/mL) and centrifuged for up to six cycles at the speed of 3000× *g* for 2′ (**a**) or 100× *g* (**e**) for 10′. The eluted protein suspension was reloaded in the upper part of the column after every cycle. H-ferritin nanocage (HFn) recovery was calculated by absorbance reading (**b**,**e**) and endotoxin (ETX) levels have been obtained by LAL test (**c**,**d**,**f**,**g**). Results from cycles 1, 3, 4 and 6 have been inserted in graphs and labelled as c#1, c#3, c#4 and c#6, respectively. Results are reported as average ± S. D. of 6 independent experiments. Statistical significance panels c and d: ** *p* = 0.0013, *** *p* = 0.0005, °°° *p* = 0.0001, °° *p* = 0.0013, ### *p* = 0.006, ^^^ *p* = 0.0002. Statistical significance panels f and g: * *p* = 0.0122, ** *p* = 0.0029, °° *p* = 0.0030, ° *p* = 0.0130, ## *p* = 0.0029, ^^. *p* = 0.0030.

In order to achieve an injectable HFn preparation, we first tried to remove ETX exploiting an affinity chromatography. Among different kinds of resins commercially available which exploits affinity adsorbents, such as immobilized l-histidine, poly- l-lysine, poly(γmethyl l-glutamate), and polymyxin B, we have chosen to test Endotrap HD, which is based on a bacteriophage-derived protein. Unlike l-histidine, poly- l-lysine, poly(γmethyl l-glutamate)-based resins which display a charge-mediated reversible interaction, Endotrap is similar to a polymixin B resin since exhibits a specific protein interaction without the toxicity observed in this latter [33,41]. Indeed, it captures the conserved region of the inner core of lipopolysaccharides molecules, and it is able to bind to all kinds of ETXs from Gram-negative bacteria. Moreover, it seems to display more stable performance at a wide variety of conditions (pH and ionic strength/salt concentration) in comparison to other kinds of ETXs-affinity resins. To assess the suitability of this resin to bind and remove ETXs from HFn, we have used Endotrap HD 1 mL columns, which are pre-packed columns useful for small scale purifications. According to the manufacturer instructions, we loaded in the column bed 3 mL of HFn at the selected concentration of 1 mg/mL and we incubated for 2 h under gravity flow. The protein recovered at the end of the incubation was near to 0% (data not shown), since almost all the protein was trapped into the column resin, as a consequence of the high molecular weight of HFn. To evaluate the capability of Endotrap HD resin to remove ETXs from HFn solution, we have forced HFn flow through the resin, centrifuging the column for 2′ at 3000× *g.* As a result of that, we have obtained a protein recovery of more than 80% (Figure S1a). We then measured the ETX level of the eluted protein obtaining 1.7 × 10^4^ EU/mL. As compared to the initial value of 9.3 × 10^5^ EU/mL, this already corresponds to a 99% reduction in the ETX content (Figure S1b). However, it is still far from the 1 EU/mL needed for pharmacological application. This result, despite obtained in small scale and forcing elution with centrifugation, which is not a process supported by manufacturers, suggests that this kind of resin exploits ETX-resin interaction suitable to segregate ETXs from HFn. To test the hypothesis that increasing the contact time between resin and HFn could be the right way to further reduce ETXs amount in HFn samples, we have tried to modify centrifugation cycles (up to six), speed and time, as described in Figure 1a.

The protein recovery obtained with the 3000× *g* centrifuge cycles varied between 86% (after the first centrifuge; c#1) and 82% (after the last centrifuge; c#6 Figure 1b). These values were higher than the ones obtained with the 100× *g* ones, which ranged between 71 and 58%, suggesting that the higher centrifugation speed allowed the protein to elute more efficiently through the column (Figure 1e). On the other hand, ETXs were more efficiently removed with the 100× *g* series of centrifuges, with values ranging between 102 and 129 EU/mL after the fourth (c#4) and sixth (c#6) centrifuge cycles (Figure 1f), as compared to 3362 (c#4) and 1263 EU/mL (c#6) for the 3000× *g* centrifuges (Figure 1c). By combining EU/mL with protein recovery, we calculated the EU/mg of protein. With the milder centrifuge cycles, we obtained 151 EU/mg of protein (Figure 1g), as compared to the ten times higher 1460 EU/mg obtained with the higher centrifugations (Figure 1d). In fact, even if we obtained a cleaner protein as compared to the single centrifuge cycle, we were not able to clean the protein below an ETXs concentration of around 10^2^ EU/mL, where it seemed to reach a plateau. This probably corresponded to the concentration of ETXs that saturated the resin.

#### 3.1.2. Batch Mode

**Figure 2:**
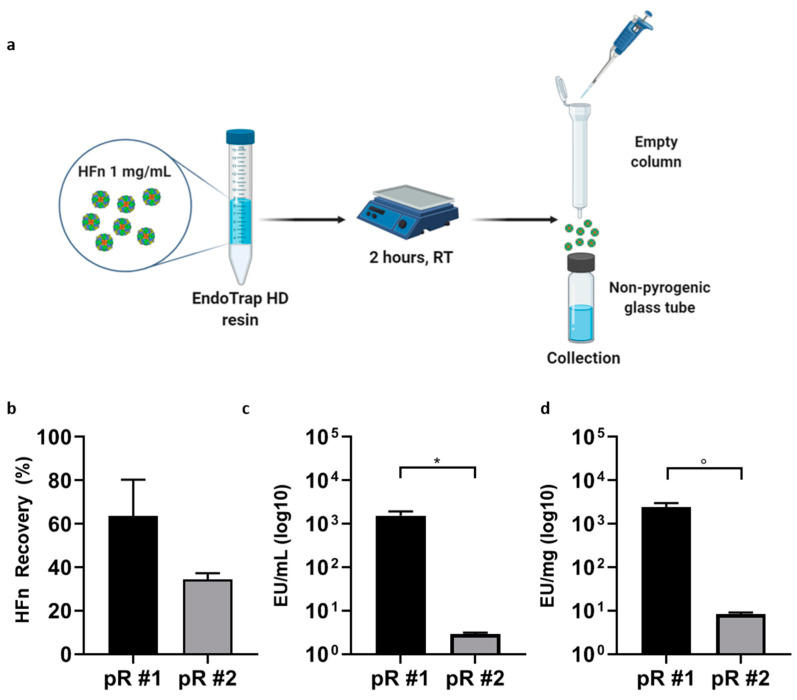
Purification Strategy using Endotrap HD resin in batch mode. HFn (1 mg/mL) was incubated with the resin for 2 h at room temperature (RT) and eluted using an empty column, where the resin was trapped (**a**). Incubation has been repeated once or twice, with new resin (pR#1 and pR#2 respectively). HFn recovery after one (black) or two cycles of incubation with resin (gray) was calculated by absorbance reading (**b**); ETX levels have been obtained by LAL test (**c**,**d**). Results are reported as average ± S. D. of 4 independent experiments. Statistical significance: * *p* = 0.0314, ° *p* = 0.0315.

In the attempt to increase the efficiency of Endotrap HD in removing ETX from HFn, thus avoiding resin centrifugation steps that might compromise resin and HFn stability, we had to tune the amount of Endotrap HD resin used for HFn purification and increase contact time. Therefore, we moved to work in batch mode. In the batch mode protocol, illustrated in Figure 2a, we incubated the protein with the resins for 2 h and we separated the cleaned protein from the resin using an empty column. This allowed us to trap the resin and the ETXs in the column and collecting the eluted protein in a non-pyrogenic tube. To perform this step, we worked in one hundred-fold excess of resin, which is calculated considering the binding capability of the resin per mL and the amount of ETX in the starting HFn sample. The protein recovery was in line with the ones obtained with the column mode, without introducing any stress due to the centrifugation steps, as demonstrated by Transmission Electron Microscopy images (TEM) reported in Figure S2b. Moreover, ETX removal efficiency was 10 times higher with the batch mode than with the column mode (10^4^ EU/mL for column mode vs. 10^3^ EU/mL for the batch mode after the first centrifugation or incubation cycle respectively). This suggests that tuning the amount of resin allowed by the batch mode is a significant advantage in terms of ETXs removal efficiency. This was confirmed as we obtained an ETX level of only 2 EU/mL after the second incubation cycle (Figure 2c), a value that was two orders of magnitude lower than what observed in the column mode. However, the drawback of this approach was that the protein recovery after the second incubation cycle was lower as compared to the column mode (34 and 59%) (Figure 2b). This might happen as in the batch mode we did not include any centrifugation step, to preserve the protein from further stress, and some of it could be left trapped in the resin.

### 3.2. Endotoxin Removal with Triton X-114

**Figure 3:**
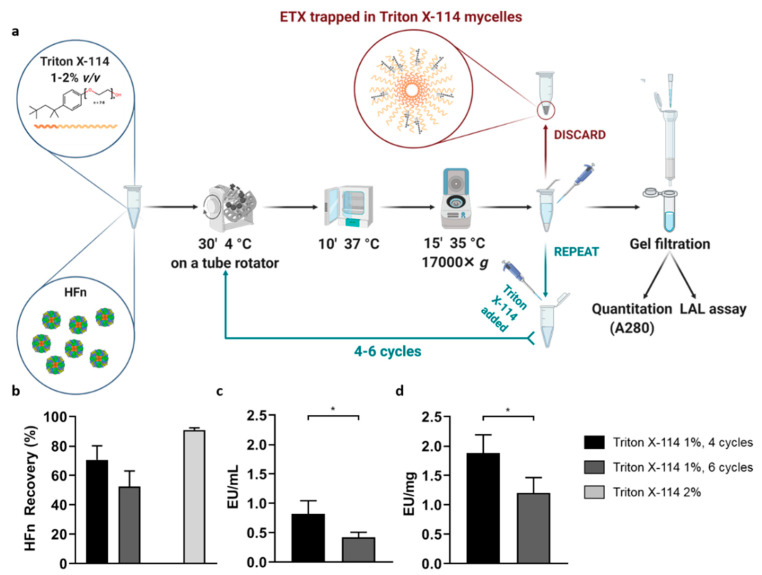
Purification strategy using Triton X-114. 1% of Triton X-114 has been added to HFn (1 mg/mL) and processed for 4 (black) or 6 (dark gray) purification cycles consisting of 30′ incubation at 4 °C followed by 10′ at 37 °C and centrifugation, as reported in panel (**a**). Also, a treatment with a single cycle of 2% Triton X-114 has been assessed (light gray). HFn recovery in each condition was calculated by absorbance reading (**b**); ETX levels have been obtained by *Limulus Amebocyte* Lysate (LAL) test (**c**,**d**). Results are reported as average ± S. D. of 6 independent experiments. Statistical significance: panel c: * *p* = 0.0263; panel d: * *p* = 0.0201.

In the attempt to increase protein recovery, we have also explored an alternative strategy to the use of resins for ETX removal, represented by Triton X-114, a non-ionic detergent with reversible phase-separation properties [38]. As reported in Figure 3a, if dissolved in water-based solvents and kept at temperatures near to 0 °C, Triton X-114 is stable in solution and forms micelles with a strong affinity for ETX. If the solution is warmed up to 37 °C, the solubility of the detergent decreases and it creates a detergent phase on the bottom of the vial, co-precipitating the attached ETX. Here, we optimized a protocol that was applied in literature to purify β-lactoglobulin [39]. We performed 4 and 6 cycles of purification using a 1% Triton X-114 solution in PBS. As it can be seen in Figure 3b, protein recovery was higher when stopping after 4 cycles of purification (65%; black), while it decreased to less than 45% after 6 cycles (dark gray). In both cases, ETX content after purification was below the 1 EU/mL recommended by the pharmacopoeia for parenteral injection, thus suggesting the reliability of this approach. A stringent requirement to validate this protocol is removing all the Triton X-114 from the HFn solution completely, as residual detergent could be toxic for cells and tissues. To obtain this goal we have performed a gel filtration on G-25 Sephadex. Evidence obtained by A280, Bradford Assay, LAL test and TEM (Figures S2 and S3) confirmed that, thanks to the sequential precipitation steps and the final gel filtration, the detergent was completely removed. Then, we tried to purify HFn with a 2% Triton X-114 solution, with the idea of decreasing the number of cycles needed to remove ETX (Figure 3b; light grey). However, after the first cycle of incubation at 2%, we were not able to remove the detergent, so we dropped this protocol without measuring ETX concentration. In fact, we showed that traces of the detergent impair LAL measurements, interacting with the LAL lysate and invalidating the results (Figure S3).

**Figure 4:**
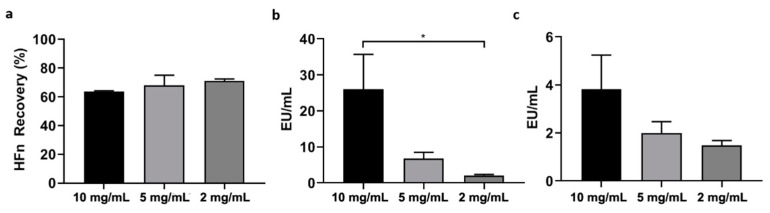
Scale-up of Triton X-114. 1% Triton X-114 has been added to HFn at different concentrations (2, 5 and 10 mg/mL) to remove ETX through 4 incubation cycles, as reported in panel a. HFn recovery was calculated by absorbance reading (**a**), while ETX levels have been obtained by LAL test (**b**,**c**). Results are reported as average ± S.D. of 4 independent experiments. Statistical significance: * *p* = 0.0487.

Based on the results obtained with HFn at a concentration of 1 mg/mL, the 4-cycle incubation with Triton X-114 appears to be the most efficient method to remove ETX. Using this protocol, we increased HFn concentration at 2, 5 and 10 mg/mL, and measured protein recovery and ETX content at the end of the procedure (Figure 4). The protein recovery was in line with the values obtained with 1 mg/mL solution (71%, 71%, 68%, 64% for 1, 2, 5 and 10 mg/mL respectively), suggesting that even at high protein concentrations, HFn stability is not impaired by the presence of the detergent, as further evidenced by TEM (Figure S2c). We measured the ETX concentration at the end of the process obtaining 2.1 EU/mL, 6.7 EU/mL and 26 EU/mL for the 2, 5 and 10 mg/mL suspensions, as compared to the 0.8 EU/mL obtained with the 1 mg/mL sample. In terms of EU/mg the values are not as dissimilar from the 1 mg/mL sample, as we measured 1.5, 2 and 3.8 EU/mg for the 2, 5 and 10 mg/mL, respectively (Figure 4c) as compared to the 1.8 for the 1 mg/mL sample. These data confirmed that, even if four cycles of Triton X-114 were not enough to reach the 1 EU/mL required threshold, this protocol can be used to purify highly concentrated HFn without incurring in protein loss and obtaining an extremely high purification yield.

### 3.3. Combination of Endotrap HD Resin and Triton X-114

**Figure 5:**
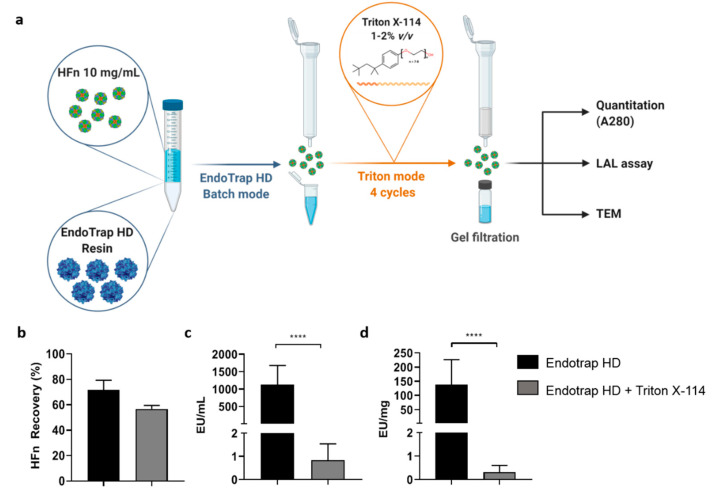
Combined Endotrap HD and Triton X-114 purification. Endotrap HD resin was incubated for 2 h with HFn (10 mg/mL; 1:1 (*v/v*) ratio) and eluted using an empty column, as described for batch mode in Figure 2a. The resulting HFn has been supplemented with 1% Triton X-114 and treated for 4 cycles of purification (**a**). HFn recovery was calculated by absorbance reading (**b**), while ETX levels have been obtained by LAL test (**c**,**d**). Results are reported as average ± S. D. of 6 independent experiments. Statistical significance: **** *p* < 0.0001.

In order to go below the 1 EU/mL ETX threshold in the highly concentrated HFn, we tried to combine the two methods tested so far, the affinity chromatography with Endotrap HD resin and detergent purification with Triton X-114 (Figure 5a). We first incubated 10 mL of a 10 mg/mL HFn solution in equilibration buffer with a 1:1 *v/v* resin for 2 h in batch mode. From a starting ETX concentration of 7 × 10^6^ EU/mL, this process allowed us to obtain a protein solution with an ETX concentration of 1124 EU/mL and a recovery of 71% of HFn (Figure 5b,c), in line with what previously obtained with the lower concentrated 1 mg/mL samples (Figure 2b,c). Then, we started the four cycles of Triton X-114 incubation, followed by the final gel filtration. The final recovery of the protein decreased to 57%, but the purification rate was exceptionally high, with a final ETX concentration of 0.83 EU/mL, corresponding to 0.32 EU/mg of protein (Figure 5b–d). This protein can be now safely used for in vitro and in vivo drug delivery applications in full respect of the pharmacopoeia limitations. Also, in this case P-NC integrity is preserved as demonstrated by TEM (Figure S2).

### 3.4. LPS Free HFn Binds and Internalize in Target Cells via TfR1 Interaction

**Figure 6:**
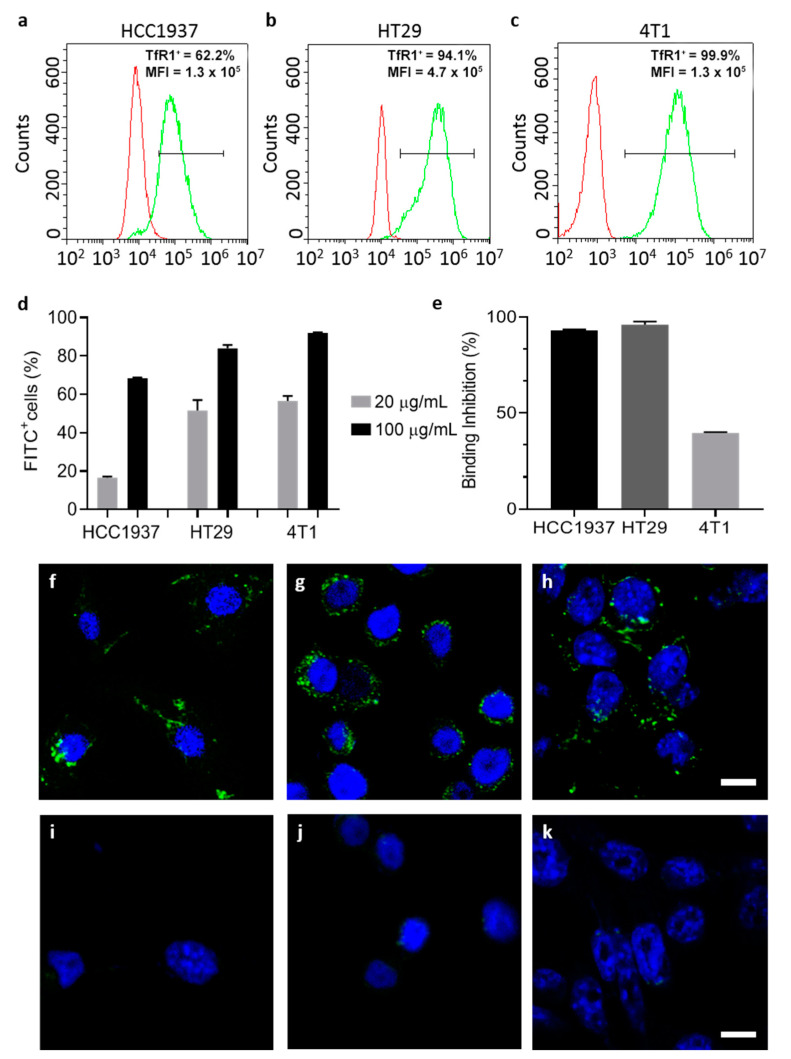
Cell interactions and uptake of ETX-free HFn. Representative histograms of TfR1 expression of selected cells (HCC1937, HT29 and 4T1) studied by flow cytometry (**a**–**c** respectively) (TfR1^+^: percentage of TfR1 positive cells, after setting proper fluorescence fates on non-stained cells; MFI: mean fluorescence intensity). Cells have been incubated with F-HFn at different concentrations at 4 °C for 2 h and binding has been evaluated by flow cytometry showing that binding is correlated with TfR1 expression (**d**); competition assay showed reduced binding of HFn FITC when cells were pre-incubated with non-labelled HFn, confirming the TfR1 binding specificity (**e**); results in panels d and e are reported as average ± S.D. of 3 independent experiments. Confocal microscopy micrographs showing uptake of ETX-free F-HFn in HCC1937, HT29 and 4T1 after incubation with 100 µg/mL of particles for 6 h (**f**–**h**). Cells were incubated with the same concentration of free FITC for 6 h and the signal was almost undetectable (**i**–**k**) F-HFn and FITC are represented in green, while nuclei labelled by DAPI are colored in blue. Scale bar 10 µm.

Since HFn is a promising tumor-targeted drug delivery agent that exploits HFn specific interaction with TfR1, we studied whether ETX purification influenced HFn binding with TfR1. To this aim, we studied binding and uptake of ETX-free HFn in three different cell lines, depending on their TfR1 expression level. We used HCC1937, HT29 (human-derived epithelial breast and colon carcinomas, respectively) and 4T1 (murine TNBC model). We verified TfR1 expression by flow cytometry, confirming that HCC1937 had lower expression than HT29 and 4T1, with 62%, 94% and 99% of positive cells respectively (Figure 6a–c). We incubated FITC labelled HFn (F-HFn, Table S1) at two different concentrations (20 and 100 µg/mL) observing a dose-dependent binding in all tested cells (Figure 6d). Moreover, the F-HFn binding was significantly lower in HCC1937, in accordance to their lower TfR1 expression (Figure 6d). To further assess if the specificity of the HFn binding (TfR1 mediated) was preserved, we performed a competition assay pre-incubating cells with an excess of non-labelled purified HFn. By doing so, subsequent binding with F-HFn was almost completely inhibited in HCC1937 and HT29 cells (with 92 and 96% binding inhibition) and significantly reduced in 4T1 cells (40% inhibition) (Figure 6e), in line with what we have previously observed with non-purified HFn [42]. These results confirm that cell binding is not influenced by the purification process and is still mediated by interaction with TfR1 surface receptors.

Finally, we evaluated uptake in cells by confocal microscopy and we found that HFn-F is efficiently internalized inside cells after 6 h of incubation. HFn is mainly distributed in small vesicle like structures typical of particle accumulation, similarly to what we previously obtained with non-purified HFn [17]. To verify that the intracellular fluorescent signal was due to an actual HFn uptake and not only to an eventual free dye diffused in cells, we incubated equivalent concentrations of free FITC. The intracellular signal observed was sensibly lower and almost undetectable as compared to the F-HFn (Figure 6i–k).

Altogether, our results show that we were able to remove ETX contamination without modifying its main properties, thus confirming that HFn is a very promising agent for drug delivery for cancer treatment.

## 4. Discussion

HFn has raised great interest in the drug delivery field and it is widely used at a preclinical level [43,44]. Several antitumor drugs currently in clinical practice have been nanoformulated in HFn nanocages to increase their therapeutic efficacy and reduce side effects [16].

Moreover, HFns have catalyzed the interest of nano-oncologists thanks to their unique properties that make them optimal for the development of new cancer therapies, such as their biocompatibility, high versatility, high control of encapsulated molecules, natural tumor homing and possibility to be functionalized on their surface. HFn specificity, in particular, is made possible since it possesses a unique feature: its natural target is TfR1, which is a physiological receptor overexpressed in several types of cancer. However, HFns are mostly obtained by self-assembling of H-chains of human ferritin produced as recombinant protein in *E. coli*. This feature brings with it the requirement to remove ETX contaminants right after its production, before proceeding with pre-clinical studies aimed to assess the suitability of every developed HFn-based nanodrug or nanotracer. Indeed, it is necessary to make HFn-based nanoformulations safe for parenteral administration and to avoid any pyrogenic response in the target organism.

So far, ETX removal was successfully performed mainly on small proteins (albumin-like or with similar molecular weights) but it was not implemented in larger, multimeric protein like HFn [37,45]. Indeed, such proteins often come with a high molecular weight (due to the presence of several subunits), thus making it harder to purify them from ETX while maintaining their quaternary structure. Therefore, in order to solve the issue, we firstly tested several commercial solutions to make the HFn as ETX-free as possible. Since we did not obtain the expected results in terms of ETX purification and protein recovery, we developed a hybrid method that proved to be easy to perform and excellent in removing ETX. Furthermore, our protocol allowed us to obtain purified HFn with good yields and, even more importantly, with high protein concentrations. That is of extreme importance because, after purification, protein nanocarriers usually undergo further processing to turn them into nanodrugs or nanotracers. Therefore, we focused our research not only on finding a good protocol to purify HFn, but we put our efforts into refining it as much as possible in order for it to be suitable for highly concentrated HFn.

Starting with currently available methods, our results showed that using ETX-binding pre-packed columns has several limitations when working with big proteins, especially if the aim is to obtain good yields while maintaining high protein concentrations. In detail, we obtained a HFn solution with a final ETX contamination that was two orders of magnitude above the requested limit, even if the protein recovery appeared to be good (about 60%). In addition, another limitation of this method was due to the impossibility of letting the protein flow by gravity, since its molecular weight (509 KDa) makes it difficult for it to pass without forcing it with one or more centrifugations, which should be avoided since such a procedure tends to stress the protein.

After testing the same resin in a batch incubation, which allowed us to increase the contact time, results were encouraging but still not optimal: two sequential purification steps allowed to purify high volumes of proteins at high concentrations with a greater decrease in ETX contaminants, but the protein recovery dropped significantly, as it was lower than 40%. Performing a single purification cycle was not optimal either, since the resulting HFn showed a good protein recovery but an ETX content that was still too high.

In an attempt to find the solution with a different approach, we then tried to purify HFn by using Triton X-114, a well-known surfactant that was successfully employed in other studies to purify small proteins. Encouraging results were obtained by using Triton X-114 with a four-cycle protocol, resulting in the removal of most of ETX (below the threshold for parenteral injection) while maintaining a very good protein recovery. However, scaling-up HFn concentration resulted in a proportional decrease of ETX removal.

An additional attempt was made by increasing the percentage of detergent (2% *v/v*) as suggested by Teodorowicz and coworkers [39]. Results obtained with HFn were inconclusive though, because such an amount of Triton X-114, even when performing a single purification step, tends to stay in solution and it becomes impossible to remove it by gel filtration. In addition to the potential toxicity of the remaining Triton X-114, its presence interferes with both quantification assays and LAL test, thus making this protocol not suitable for our purpose [46].

The solution came with the combination of the two methods (i.e., Endotrap affinity chromatography and treatment with Triton X-114), which resulted in a protocol able to effectively purify HFn for parenteral infusion with high yields. With the hybrid protocol here proposed, we successfully purified highly concentrated HFn (up to 10 mg/mL) with an average protein recovery of around 60% (which is surprisingly high for such large proteins) and incredibly high purification rates (below 1 EU/mL and far below 1 EU/mg), thus making the protein suitable for in vivo applications.

In addition, by employing this strategy, protein integrity and cell targeting functionality are retained as demonstrated by TEM and by functional assays performed with ETX-free HFn.

Upon ETX purification, TfR1-mediated interaction with cancer cell lines is maintained as demonstrated in binding and competition assays. 

Therefore, overall this new hybrid technique could possibly be applied to several protein nanocages (or just proteins with a high molecular weight and/or multiple subunits), especially when conventional methods appear to be ineffective. This protocol is easy to learn and master, requires limited skills and instruments and has the potential to make protein nanocages (produced in bacteria) ETX-free, as requested by the institutions that are responsible for the approval of new nanoformulations for in vivo applications. In addition, ETX removal with this method is not only highly reproducible but it can be easily tested via LAL assay, which is itself officially recognised as a solid test to assess the presence of ETXs in a protein sample.

Future experiments will focus on the immunogenicity of this nanocage, with the aim of proving it to be non-pyrogenic and not detectable by the immune system. The expectations about this topic are especially high, since ETX-free HFn shows the same identical characteristics of its physiological variant, and such similarity should indeed result in a complete lack of activation of the immune system, as the trigger would be ETX binding to Toll-like receptors on innate immune system cells: without ETX, purified HFn should definitely act as if it was produced by the organism itself.

In conclusion, our protocol proved to be effective in purifying recombinant HFn from extremely high initial concentrations of ETX, to well below the accepted limit of 1 EU/mL, making this protein suitable for in vivo preclinical application and, eventually, even clinical trials on humans. Our combined method, that can be easily done on every lab bench, couples high purity yields and high recoveries with high reproducibility and contained costs. Therefore, it can be easily used to remove ETX from all those large, multimeric, P-NCs that are being developed as nanosized drug delivery agents, bringing them one step closer to future clinical applications.

## Figures and Tables

**Table 1:** Protocol description for column mode purification with Endotrap HD 1 mL columns.

Protocol	Centrifugation Steps	Centrifugation Speed	Centrifugation Time
#1	6	3000× *g*	2′
#2	6	100× *g*	10′

## Data Availability

Data available in a publicly accessible repository https://doi.org/10.13130/RD_UNIMI/ALYI3H, after publication.

## Supplementary Materials

Supplementary Materials can be found at https://www.mdpi.com/1999-4923/13/2/229/s1. Figure S1: A solution of HFn (1 mg/mL) has been incubated in Endotrap HD 1 mL columns and then centrifuged (3000× *g*, 2’) to force the flow of the protein through the column. Protein recovery has been measured by absorbance reading (A280 nm) (a); ETX concentration of the protein before the incubation and after the centrifugation have been measured by LAL test (b).; Figure S2. TEM representative images of native HFn (a), and after purification with Endotrap HD resins (b), Triton X-114 (c) and the combined method of the two (d). Scale bar = 50 nm.; Figure S3. LAL reaction curves and Triton X-114 interference. Representative reaction plots of the standards at different ETX content (50, 5, 0.5, 0.05 and 0.005 EU/mL respectively) used in the LAL kinetic turbidimetric test to prepare the standard curve (a). The standard curve (b) is made by plotting on the x axis the time (minutes) when the turbidity of each one of the standards reaches an absorbance of 0.1 (dashed line of panel a), and on the y axis the relative ETX concentration. Interference of high concentrations of Triton X-114 (1%) in LAL test turbidimetric readings, as shown in a sample of HFn purified with 6 cycles of Triton X-114 before G25 detergent removal (empty squares); Triton X-114 interference can be observed also at low concentrations (0.1%), where it inhibits the reaction of the spike 5 EU/mL (red empty triangles) as compared with the spike in PBS alone (green empty circles) (c). Representative turbidimetric plots of HFn purified with 6 cycles of Triton X-114 where the detergent has been removed by G25 columns (empty triangles). The plot of the spike (empty rotated squares) overlaying the 5 EU/mL standard in PBS, confirms the successful removal of the detergent (d).; Table S1. HFn FITC characterization.
