# Supplementary material for: Combined Method to Remove Endotoxins from Protein Nanocages for Drug Delivery Applications: The Case of Human Ferritin
Source: Pharmaceutics. 2021 Feb 6;13(2):229. doi: 10.3390/pharmaceutics13020229 (PMC7915212; doi:10.3390/pharmaceutics13020229)
Supplement: Supplementary file 1 [file pharmaceutics-13-00229-s001.pdf]

pharmaceutics

MDPI

Supplementary Materials: Combined Method to Remove Endotoxins from Protein Nanocages for Drug Delivery Applications: The Case of Human Ferritin

Filippo Silva, Leopoldo Sitia, Raffaele Allevi, Arianna Bonizzi, Marta Sevieri, Carlo Morasso, Marta Truffi, Fabio Corsi and Serena Mazzucchelli

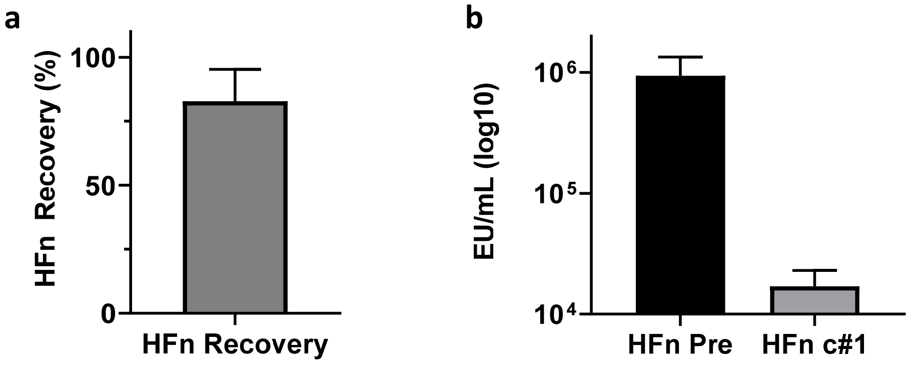

Figure S1: A solution of H- Ferritin (HFn; 1 mg/mL) has been incubated in Endotrap HD 1 mL columns and then centrifuged (3000× *g*, 2′) to force the flow of the protein through the column. Protein recovery has been measured by absorbance reading (A280 nm) (**a**); Endotoxin (ETX) concentration of the protein before the incubation and after the centrifugation have been measured by Limulus Amebocyte Lysate (LAL) test (**b**).

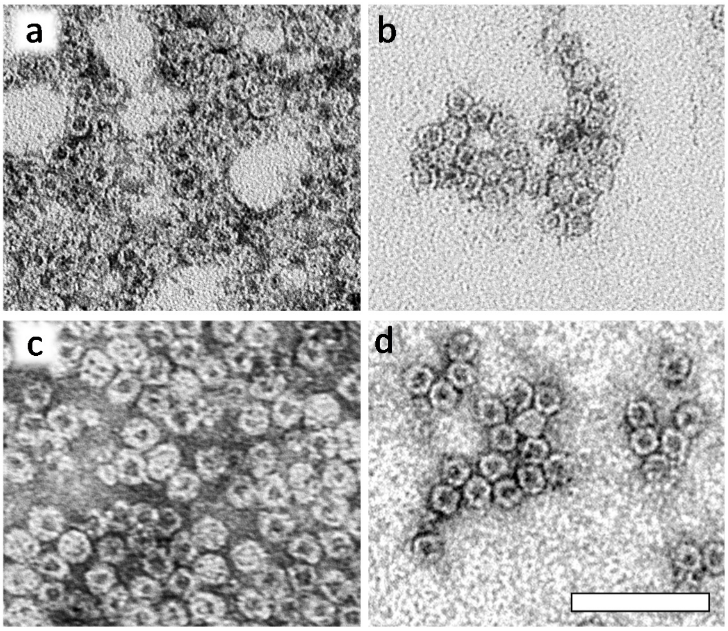

Figure S2. TEM representative images of native HFn (**a**), and after purification with Endotrap HD resins (**b**), Triton X-114 (**c**) and the combined method of the two (**d**). Scale bar = 50 nm.

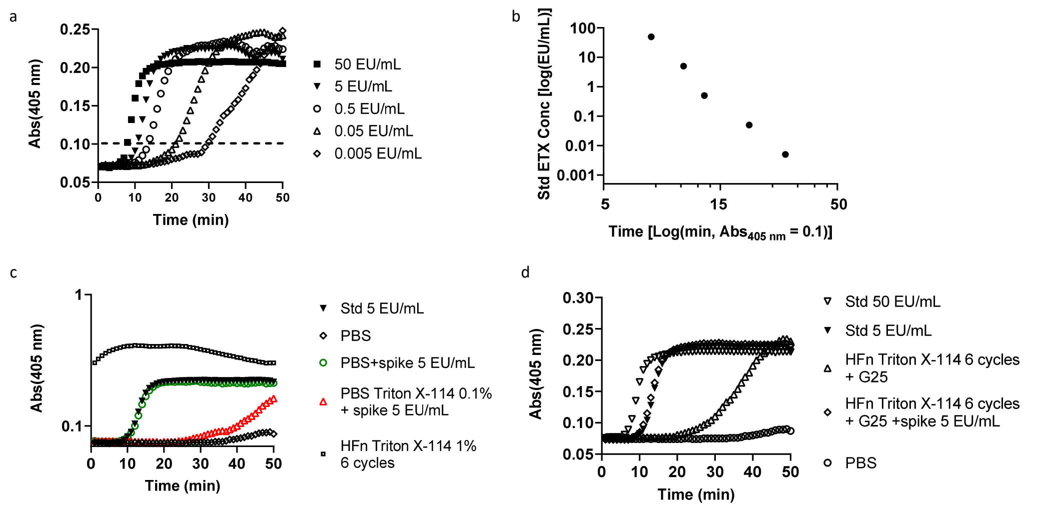

Figure S3. Limulus Amebocyte Lysate (LAL) reaction curves and Triton X-114 interference. Representative reaction plots of the standards at different ETX content (50, 5, 0.5, 0.05 and 0.005 EU/mL respectively) used in the LAL kinetic turbidimetric test to prepare the standard curve (**a**). The standard curve (**b**) is made by plotting on the x axis the time (min) when the turbidity of each one of the standards reaches an absorbance of 0.1 (dashed line (**a**)), and on the y axis the relative ETX concentration. Interference of high concentrations of Triton X-114 (1%) in LAL test turbidimetric readings, as shown in a sample of HFn purified with 6 cycles of Triton X-114 before G25 detergent removal (empty squares); Triton X-114 interference can be observed also at low concentrations (0.1%), where it inhibits the reaction of the spike 5 EU/mL (red empty triangles) as compared with the spike in PBS alone (green empty circles) (**c**). Representative turbidimetric plots of HFn purified with 6 cycles of Triton X-114 where the detergent has been removed by G25 columns (empty triangles). The plot of the spike (empty rotated squares) overlaying the 5 EU/mL standard in PBS, confirms the successful removal of the detergent (**d**).

Table S1. HFn FITC characterization.

Sample	HFn (mg/mL)	FITC (µM)	FITC µM/100 µg HFn
HFn-FITC	1.85	33.2	1.79
